# Medical Essays

**Published:** 1837-07

**Authors:** 


ART. V.—*Medical Essays.* By J. HUNGERFORD SEALY, M.D.E. A.B. T.C.D. No. I. *Phthisis Pulmonalis, curable and incurable.* No. II. *The Imagination, its history and effects.—London,* 1837. 12mo. pp. 82 and 91.

IT is with unfeigned regret that we record our opinion of these small volumes, because it may possibly give pain to their author, who, we are given to understand, is a physician highly respectable and respected, both in his private and professional capacity. But we should hold ourselves unworthy of the high office we fill, and the functions of which we have undertaken to discharge honestly, if we shrunk from recording our judgment of any book that comes before us, when circumstances seem to require that we should notice it at all. Most persons, we suspect, on reading the present work, will be disposed to set it down as the production of some youth, alike undisciplined in logic and medical science, who, undeterred by the remonstrances of his wiser and more experienced friends, and listening only to the suggestions of literary vanity, has rushed headlong to the press with the crude fruits of his first scientific studies; and, although we see, from the preface, that this is not exactly the case, we are still disposed to believe that, in a moment of weakness, Dr. Sealy has been tempted to present to the public what may have pleased his own undisciplined judgment when composed, but which ought never to have been permitted to leave the academic portfolio where it was originally deposited. We confess our surprise that any physician, acquainted with the present state of medical science in this country, should have thought of committing such a work as this to the press. It certainly would not be very easy to find in our recent literature, physiological and pathological speculations less supported by facts or reasoning than those contained in the first of these volumes; while the metaphysical and ethical lucubrations in the second, set all the principles of intellectual philosophy, old and new, and all sound logic, at defiance. The work is hardly less defective in a literary point of view; the language being incorrect and careless, and disfigured by much bad taste. Our chief reason for noticing the work is, to warn our inexperienced readers against being misled by doctrines which possess certainly one feature of attraction, *novelty*, and which are announced with some degree of pretension. The first essay is stated by the author to be "an attempt to distinguish those forms of phthisis pulmonalis which are curable from those which are incurable," and to contain "a novel view of scrofula and tubercular depositions;" and, in the second, he "trusts that the views which he has set forth of the great powers of the imagination, and the importance of its culture and direction, will find an echo in the bosoms of each and all of his readers."

